# Influence of Spine-Focused Verbal Instruction on Spine Flexion During Lifting

**DOI:** 10.2478/hukin-2022-0085

**Published:** 2022-11-08

**Authors:** Nevinn W. Becker, Amber D. Ziebarth, Dennis J. Larson, Derek P. Zwambag, Stephen H. M. Brown

**Affiliations:** 1 Department of Human Health and Nutritional Sciences, University of Guelph, Guelph, ON, Canada.

**Keywords:** trunk, bending, injury, kinematics, intervertebral

## Abstract

Lifting with a flexed spine, especially near the end range of motion, has been identified as a potential risk factor for low back injury/pain. Therefore, individuals who develop discomfort from repetitive, prolonged and/or loaded flexed or slouched postures may benefit from a greater awareness of how to control and/or modify their spinal posture to avoid irritating their backs in these situations. This study was therefore designed to test the ability of spine-oriented verbal instructions to reduce intersegmental spine flexion during three lifting tasks. The lifts were first performed without any instructions on lifting technique. An audio recording was then played with instructions to limit bending in the lower back before repeating the lifts. Following the verbal instructions, maximum spine flexion angles significantly (p < 0.05) decreased at intersegmental levels in the lower thoracic and upper lumbar (T8/T9 to L2/L3) regions, but no significant changes were observed at the lower lumbar levels (L3/L4 to L5/S1). Thus, it is concluded that spine-oriented verbal instructions can decrease spine flexion during lifting; however, other cues/instructions may be required to target lower lumbar levels which have been identified as the most prone to injury/pain.

## Introduction

Epidemiological reports have identified manual lifting as a risk factor for the development of low back pain (LBP) and/or injury (Burdof and Sorock, 1997; Ferguson and Marras, 1997; Kelsey et al., 1984). Mechanistically, both the posture of the low back and the forces acting on the low back can interact to elevate this risk. One aspect of posture that modulates the risk of injury is the magnitude of trunk flexion (Hoogendoorn et al., 2000; Marras et al., 1997; Punnett et al., 1997). There are several possible biomechanical explanations for this elevated risk. Repetitive flexion and flexing with a heavy load, toward the end range of motion, create high stresses on the posterior elements of the intervertebral disc; tissue-based experiments have shown that this can cause disc herniations (Callaghan and McGill, 2001; Veres et al., 2009; Wade et al., 2014). Furthermore, when in a flexed posture, the orientation of the erector spinae musculature changes such that their fibres become more aligned along the spine’s compressive axis (Harriss et al., 2015, McGill et al., 2000), impairing their ability to counteract anterior shear forces which may cause injury (Norman et al., 1998; Skrzypiec et al., 2016; van Dieen et al., 2006; Yingling et al., 1999). Even without consideration for muscle forces, anterior shear failure force is lower in a flexed posture compared to a neutral posture (Howarth et al., 2012). Additionally, the incidence and re-aggravation of LBP are commonly reported in movements requiring trunk flexion (O’Sullivan, 2000). Therefore, individual knowledge of how to reduce or limit the magnitude of flexion used during these tasks is of potential value. Finally, those who develop discomfort from repetitive, prolonged and/or loaded flexed or slouched postures may benefit from a greater awareness of how to control and/or modify their spinal posture to avoid irritating their backs in these situations.

Various methods have been proposed to attempt to change motion, including tactile cues (Martin et al., 2015; Pinto et al., 2018), auditory feedback (Kamachi et al., 2021), and verbal instructions (Beach et al., 2018). A simple verbal instruction seems to be the simplest method to implement and is used in workplaces requiring manual labor (van Dieen et al., 1999). Moreover, a study conducted by Beach et al. (2018) found that instructions using spine-oriented language could effectively decrease the amount of spine flexion used during a controlled box lift. More specifically, spine-oriented language was more effective than giving no verbal instruction, and it was more effective than the common verbal instruction to “lift with your legs instead of your back” (Beach et al., 2018). The kinematic spine measure in the Beach et al. (2018) study was the angle of the thorax with respect to the pelvis, which encompasses the entire lumbar spine. Recently, a method was developed by Zwambag et al. (2018) which enables the approximation of spine intersegmental angular kinematics by tracking the skin surface curvature. This is important as initial provoking damage and degeneration may occur at individual intervertebral levels (Luyendijk, 1987)), and instability is generally considered to be a segmental phenomenon (Panjabi, 1992). Nonetheless, the intersegmental distribution of spine motion during functional tasks, how spine-oriented instructions affect intersegmental motion, and whether these instructional effects can be translated to lift-types performed in everyday life remain unexplored.

Therefore, the purpose of this study was to: (1) quantify the magnitude of flexion naturally achieved relative to the individual’s maximum at each intersegmental level from T8/T9 to L5/S1 during the following functional lifting tasks: picking up a pencil (PP), picking up a box freely (FB), and picking up a box over a mid-thigh barrier (BB), and (2) observe how verbal instructions to avoid lumbar spine flexion affected each intersegmental level during these lifting tasks. It was hypothesized that instructions to limit flexion through the lower back using spine-centered language would significantly decrease flexion angles at each intersegmental level evaluated.

## Methods

### Participants

A sample of sixteen healthy individuals (8 males; 8 females, mean ± (SD) age 22.6 (6.0) years, body mass 75.3 (14.7) kg, body height 175.1 (2.7) cm), volunteered to participate in the study. Exclusion criteria included history of low back pain within the previous year, current pain or discomfort that could alter natural movement, or allergies to adhesives. All participants provided informed consent. The University’s Research Ethics Board approved the procedure used in this study.

### Design and Procedures

**Figure 1:**
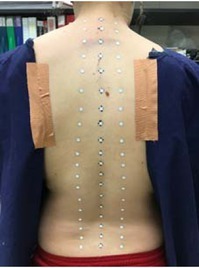
An example of the intersegmental spine kinematic marker set-up. The middle column was placed over spinous processes from vertebral levels C7 to S1, while the lateral columns were situated laterally by 3 to 5 cm over the apex of the erector spinae musculature.

Male participants were asked to be shirtless throughout the entire protocol, while females were able to choose between two options: (1) wearing a lab provided backless dress with the bra unhooked and taped to the skin, or (2) wearing a sports bra. All eight females chose the backless dress option. A total of 57 circular passive reflective markers (6.5 mm diameter) were situated in three columns along the participant’s back. The middle column was placed on the skin over each spinous process from vertebral level C7 to S1, and each adjacent column was placed roughly 3 to 5 cm lateral to the spinous processes on the apex of the erector spinae musculature, providing a reflective grid of 3 columns and 19 rows (Figure 1).

Raw spine kinematic data were collected at a sampling frequency of 120 Hz using Optitrak Prime 13W cameras (Optitrak, Natural Point, Inc., Coravallis, OR, USA). A custom intersegmental spine model was used to calculate angular displacements between each adjacent spine level. This was done by applying a piecewise function using five knots and six segments to fit the X, Y, and Z position of each column of markers into a cubic polynomial as a function of the spine level. This produced a 3D spline for each column of markers. Local coordinate systems were created orthonormal to each spine level from C7 to S1, where the Superior/Inferior (SI) vectors were tangent to the middle spline, the Anterior/Posterior (AP) vector was perpendicular to the SI vector and the vectors connecting the left and right splines, and the Medial/Lateral (ML) vector was perpendicular to the SI vector and the AP vector. An in-depth description can be found in Zwambag et al. (2018). For the purposes of this study, flexion angles were analyzed at each intersegmental level from T8/T9 to L5/S1 as well as the full lumbar regional angle from T12 to S1. Axial twist angles were also analyzed for the non-sagittal plane PP task for the same levels as the flexion angles. Angles were then filtered at a cutoff frequency of 2 Hz using a zero-lag 4_th_ order low pass Butterworth filter. All data analysis was completed using MATLAB (The Mathworks, Natick, MA, USA).

**Figure 2:**
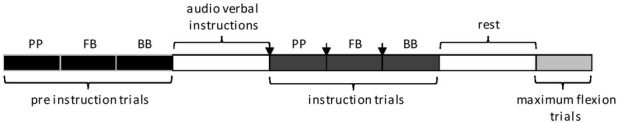
A schematic depiction of the protocol. Please see the Design and Procedures subsection of the Methods for specific details. PP = pencil pickup, FB = free box lift, BB = box lift over a barrier.

Participants performed three trials of each of three functional lifting tasks in the following order: pencil pick-up (PP), free box lift (FB), and box lift over a barrier (BB). All three lifting tasks started and ended in upright standing. The box had handles 15 cm above the ground. Barrier height was set to the mid-point between each participant’s patella and greater trochanter, and it was placed directly anterior to each participant’s feet. Participants could familiarize themselves with the mass of each object (pencil: 0.005 kg, box: 2.5 kg) before performing the task and were advised to set the object on the ground at a distance in front of them that felt “normal” for an everyday pick-up; this was to mitigate any learning effect between trials and promote natural movement. Once all pre-instruction lifting tasks were completed, participants were given two to four minutes to relax and move around the lab. Then an approximately 40 s audio recording was played for each participant focusing on three items: 1) it asked participants to focus on how much they round their lower back during the subsequent lifts; 2) it provided a statement that regularly approaching maximum spine flexion during lifting has been identified as a risk for developing low back pain or injury; 3) it told participants that the way they choose to move should feel natural and not robotic or awkward. After listening to this audio recording, the participant repeated the lifting tasks, with additional audio recorded verbal instruction provided between each set of lifts to limit the amount that they rounded or bent their lower back as they moved. This occurred as follows: audio verbal instruction, PP (3 trials), audio verbal instruction, FB (3 trials), audio verbal instruction, BB (3 trials) (Figure 2). The audio recorded verbal instruction was re-played at any time if requested by the participant. After these post-instruction lifting tasks were completed, participants were again given two to four minutes to relax and move around the lab. Finally, participants performed three maximum spine flexion trials, conducted with the pelvis constrained and instructions to flex their spine as far as possible. The maximum flexion trials were used to normalize the flexion angles measured during the lifting trials, allowing for them to be expressed as a percentage of their maximum flexion.

### Statistical analysis

The peak flexion angle as a percentage of maximum was tested for each of the three lifting tasks using a two-way repeated measures ANOVA at each intersegmental level as well as the lumbar region. The independent variables were condition (No Instructions, Instructions) and sex (Male, Female). The absolute (degrees) peak flexion angle was tested for each of the three lifting tasks using a one-way repeated measures ANOVA at each intersegmental level as well as the lumbar region. The independent variable was condition (No Instructions, Instructions). The absolute axial twist angle was additionally examined using a two-way repeated measures ANOVA for the PP condition, as it inherently produced non-sagittal plane motion. The level of significance was set at *p* < 0.05. All statistical analyses were performed using SAS University Edition (SAS Institute, Cary, NC, USA).

## Results

### Percentage of maximum flexion

**Figure 3:**
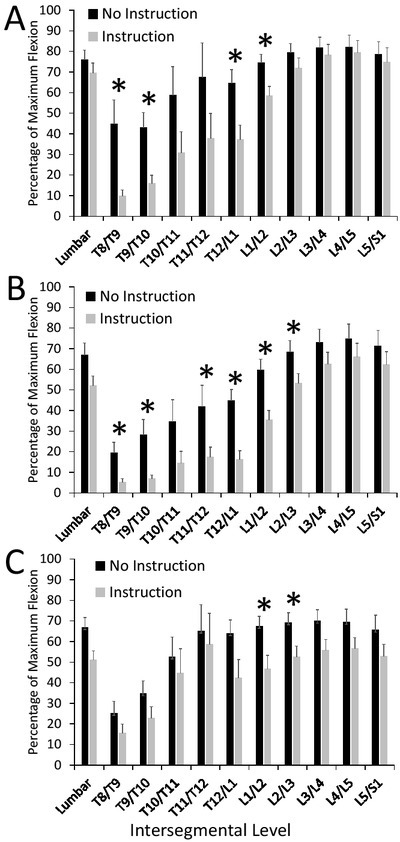
Mean (+SEM) peak percentage of maximum flexion angles in the gross lumbar region as well as at each intersegmental level from T8/T9-L5/S1 observed in the Pencil Pickup (A), Free Box Pickup (B), and Box with Barrier Pickup (C) tasks with no instructions (“No Instruction”) and after instructions (“Instruction”). Asterisks (*) indicate statistically significant differences between conditions (p < 0.05).

The verbal instructions elicited similar changes in all three tasks. In the PP task (Figure 3A), the % maximum flexion significantly decreased at T8/T9 (*p* = 0.0088; effect size (Cohen’s *d*) = 1.2), T9/T10 (*p* = 0.0029; effect size = 1.2), T12/L1 (*p* = 0.0067; effect size = 1.0), and L1/L2 (*p* = 0.0149; effect size = 0.9) following verbal instructions. For the FB task (Figure 3B), significant decreases in the % of the maximum flexion angle of T8/T9 (*p* = 0.016; effect size = 1.0), T9/T10 (*p* = 0.012; effect size = 1.1), T11/T12 (*p* = 0.049; effect size = 0.8), T12/L1 (*p* = 0.0003; effect size = 1.4), L1/L2 (*p* = 0.0015; effect size = 1.2), and L2/L3 (*p* = 0.0471; effect size = 0.7) were observed. The BB task (Figure 3C) showed significant decreases in the % of maximum flexion in the gross lumbar region (*p* = 0.025; effect size = 0.9), as well as at L1/L2 (*p* = 0.017; effect size = 0.9) and L2/L3 (*p* = 0.031; effect size = 0.8). There was no statistically significant changes observed at L3/L4, L4/L5, or L5/S1 for the PP task (all *p*-values > 0.66), the FB task (all *p*-values > 0.23), or the BB task (all *p*-values > 0.076).

There was no significant difference in the % of maximum flexion used between males and females during each task and males did not respond differently than females to instructions (i.e., there were no statistically significant interaction effects between instructions and sex).

### Absolute flexion (degrees)

In the PP task, verbal instructions caused a significant decrease in the absolute flexion angle at T8/T9 (*p* = 0.0002), T9/10 (*p* = 0.0067), and T12/L1 (*p* = 0.0462) (Table 1). In the FB task, verbal instructions caused a significant decrease in the absolute flexion angle in the gross lumbar region (*p* = 0.0281), T8/T9 (*p* = 0.0109), T9/T10 (*p* = 0.0123), T10/T11 (*p* = 0.0175), T12/L1 (*p* = 0.0175), L1/L2 (*p* = 0.0098), and L2/L3 (*p* = 0.0442) (Table 1). In the BB task, verbal instructions did not cause any significant changes in the absolute flexion angle at any regional or intersegmental level (Table 1).

**Table 1:** Mean (SEM) absolute degrees of flexion observed in the gross lumbar region and at each intersegmental level from T8/T9-L5/S1 for the Pencil Pickup (PP), Free Box Pickup (FB) and Box with Barrier Pickup (BB) tasks. Effect sizes (Cohen’s d) are also shown.

*PP Lift*	No Instruction	Instruction	Statistical Significance	Effect Size (Cohen’s *d*)
Region	*°Flexion*	*°Flexion*		
Lumbar	41.4 (2.9)	37.2 (2.2)		0.4
T8/T9	0.9 (0.1)	0.3 (0.1)	*	1.5
T9/T10	1.2 (0.2)	0.4 (0.1)	*	1.1
T10/T11	1.7 (0.4)	0.8 (0.2)		0.7
T11/T12	2.3 (0.5)	1.2 (0.4)		0.6
T12/L1	3.4 (0.5)	2.0 (0.4)	*	0.7
L1/L2	5.3 (0.4)	4.1 (0.4)		0.7
L2/L3	7.4 (0.5)	6.6 (0.4)		0.4
L3/L4	10.1 (0.7)	9.5 (0.6)		0.2
L4/L5	10.8 (0.9)	10.1 (0.7)		0.2
L5/S1	5.4 (0.6)	5.0 (0.4)		0.2
*FB Lift*				
Lumbar	35.6 (2.6)	28.0 (2.0)	*	0.8
T8/T9	0.4 (0.1)	0.1 (0.0)	*	1.0
T9/T10	0.8 (0.2)	0.2 (0.1)	*	1.1
T10/T11	0.9 (0.2)	0.4 (0.1)	*	0.9
T11/T12	1.4 (0.3)	0.6 (0.2)		0.7
T12/L1	2.4 (0.4)	1.0 (0.3)	*	0.9
L1/L2	4.1 (0.4)	2.6 (0.4)	*	1.0
L2/L3	6.3 (0.5)	5.0 (0.4)	*	0.7
L3/L4	8.8 (0.7)	7.6 (0.6)		0.5
L4/L5	9.5 (0.9)	8.4 (0.7)		0.3
L5/S1	4.8 (0.5)	4.2 (0.4)		0.3
*BB Lift*				
Lumbar	37.2 (3.4)	29.2 (4.0)		0.5
T8/T9	0.7 (0.1)	0.4 (0.1)		0.6
T9/T10	1.1 (0.2)	0.7 (0.2)		0.6
T10/T11	1.8 (0.4)	1.2 (0.3)		0.4
T11/T12	2.5 (0.5)	1.8 (0.4)		0.4
T12/L1	3.3 (0.4)	2.3 (0.5)		0.6
L1/L2	4.8 (0.4)	3.5 (0.5)		0.7
L2/L3	6.5 (0.5)	5.1 (0.7)		0.6
L3/L4	8.8 (0.9)	7.2 (1.0)		0.4
L4/L5	9.3 (1.1)	7.7 (1.1)		0.4
L5/S1	4.5 (0.6)	3.7 (0.6)		0.4

Asterisks (*) indicate statistically significant differences between instruction and no instruction conditions (p < 0.05).

In the non-sagittal plane PP task, the verbal instructions did not elicit any change in the magnitude of the maximum axial twist (all *p*-values > 0.14; in the lumbar region all *p*-values > 0.68) (Table 2), and there were no statistically significant interactions between instructions and sex (all *p*-values > 0.46).

**Table 2:** The mean (SEM) absolute degrees of the axial twist observed in the gross lumbar region and at intersegmental levels from T8/T9-L5/S1 for the Pencil Pickup (PP) task.

*PP Task*	No Instruction	Instruction
Region	*°Axial Twist*	*°Axial Twist*
Lumbar	5.1 (0.8)	5.4 (0.8)
T8/T9	1.0 (0.2)	1.2 (0.2)
T9/T10	1.0 (0.2)	1.3 (0.2)
T10/T11	1.1 (0.2)	1.4 (0.2)
T11/T12	1.1 (0.2)	1.4 (0.1)
T12/L1	1.1 (0.2)	1.2 (0.2)
L1/L2	1.1 (0.2)	1.2 (0.2)
L2/L3	1.1 (0.2)	1.1 (0.2)
L3/L4	1.0 (0.2)	1.1 (0.2)
L4/L5	0.9 (0.2)	1.0 (0.1)
L5/S1	0.9 (0.2)	0.8 (0.1)

No statistically significant differences were found between the instruction and no instruction conditions.

## Discussion

The first purpose of this study was to observe how close individuals naturally get to their maximum flexion at each intersegmental level during everyday lifting tasks including picking up a pencil from the ground and lifting boxes from the ground with and without barriers. In all three tasks, participants consistently reached flexion angles that exceeded 65% of their maximum range of motion in the lower lumbar intersegmental levels (L3-S1) when no verbal instructions were provided; these relative flexion angles in general became progressively lower as you moved up the spine into the upper lumbar and lower thoracic levels (Figure 3). The second purpose of the study was to determine whether spine-oriented verbal instructions could be effective in reducing spine flexion in these lifting tasks. The hypothesis that verbal instructions would decrease the flexion angle at each intersegmental level during all three lifting tasks was not supported. The only statistically significant decreases occurred at intersegmental levels between T8/T9 and L2/L3. These findings suggest that a simple verbal instruction may be effective in reducing the amount of flexion used at the lower thoracic and upper lumbar intersegmental levels during various lifting tasks; however, reducing flexion in the lower lumbar intersegmental levels will require further investigation.

The reasons why participants were able to use these verbal instructions to reduce flexion at the lower thoracic and upper lumbar levels, but not the lower lumbar levels, are unclear. Swinkels and Dolan (2000) presented data that suggested that people had a poorer position sense (a marker of proprioception) at the sacrum compared to upper lumbar and lower thoracic regions; it is possible that this regionality of the position sense was reflected in the participants’ ability to respond to verbal instructions in the current study. Previous work from our lab has demonstrated that regionally applied tactile feedback, in the form of tape applied to the skin to amplify proprioceptive input, was successful in reducing flexion at all lumbar intersegmental levels (Beaudette et al., 2018). Without this added regional proprioceptive feedback, people may struggle to modify motion at their lower lumbar intersegmental levels.

In addition, the current findings demonstrate that the extent to which individuals can adapt their lifting technique and decrease spine flexion is dependent on the context of the task. For example, compared to the PP and FB lifts, the BB lift demonstrated reduced flexion at fewer intersegmental levels following verbal instructions (Figure 3 and Table 1). The use of a barrier in the BB lift was designed to limit knee flexion and thereby force participants to use relatively more spine flexion to reach and lift the box compared to the FB task. Interestingly, after receiving the verbal instructions some participants indicated a perceived difficulty of limiting flexion in their lower back during the BB lift; for example, comments from two participants included, “this is going to be hard” (participant A) and “that’s impossible” (participant B). As these comments were verbalized immediately prior to attempting the post-instruction BB, but not FB lifts, it suggests that these individuals perceived that they needed to use more lumbar flexion, and therefore would have more difficulty reducing it, during the pre-instruction BB lifts compared to the FB lifts. This perception was true for participant B, but not participant A, and the group mean data showed relatively little absolute difference in lumbar spine flexion angles between the FB and BB lifts prior to the verbal instructions (Table 1), with greater differences existing in the thoracolumbar region (T10/T11 to L1/L2). This again brings into question the quality of proprioceptive ability and perception of movement that exists in the lower lumbar region, at least in some individuals.

There are limitations in the current study design that need to be considered. First, one goal of the study was to quantify how individuals perform relatively simple lifting tasks naturally in a ‘real-world’ scenario. To this end, in the PP and FB lifts participants were allowed to place the box/pencil at a horizontal distance in front of them that felt the most comfortable and natural. By not controlling this distance, some participants may have inherently needed to use more or less spine flexion to perform the lift based on the distance they set. Although this may have increased the variability between subjects in their lifting technique used, we believe that the results provide greater external validity; participants performed the lift how they would in an unconstrained real-world scenario. Despite this, it is possible that participants lift differently in a lab-based setting compared to how they would lift naturally outside of the lab; inherent in this is the possibility that participants limited their spine flexion even prior to receiving the verbal instructions, simply because they were lifting in a lab-based setting. Second, no information regarding the participants’ background knowledge or beliefs about lifting mechanics or low back injury was recorded. It is possible that some participants may have arrived at the study with pre-set beliefs and/or lifting styles designed to limit lumbar flexion, and therefore would have been less likely to change in response to the verbal instructions. Next, it is important to be clear that the intersegmental spine angles presented here are not directly indicative of bony motion, but rather are approximated from the tracking of the spine skin-surface curvature (Zwambag et al., 2018). Using this technique, Zwambag et al. (2018) demonstrated relatively little effect of skin motion artifact and surface marker error on computed angles. Thus, while we cannot state that the intersegmental angles explicitly represent the underlying motion of the corresponding vertebral units, we are confident that they provide a fair representation of distribution of vertebral motion along the spinal column. Finally, it is unknown whether the reductions in flexion angles observed after the verbal instructions are biologically significant. It is important to reiterate that lumbar spine flexion should be considered a population-based risk factor for the development of low back pain or injury (Burdof and Sorock, 1997; Ferguson and Marras, 1997; Kelsey et al., 1984), and that many individuals who regularly flex their spines may never develop a related low back problem. Thus, the effectiveness of any reduction in flexion is going to be highly dependent on the individual. Furthermore, it is beneficial that any instructions do not instill a fear of flexion-related movement, but rather should focus on an awareness of how flexion can be controlled under certain scenarios. For individuals who have a history of flexion-related pain or aggravation, teaching awareness of how they move their spines and how to reduce and/or control the aggravating flexion could certainly be beneficial. This is also true for athletes who may benefit from additional movement degrees of freedom as they learn techniques to improve overall performance and/or to reduce discomfort in their low backs. However, the verbal instructions employed in the current study only resulted in statistically significant reductions in flexion at intersegmental levels at and above L2/L3. Therefore, continued work is necessary to understand how to improve motion awareness and invoke potential flexion reductions in the lower lumbar spine.
